# Case report: Pediatric vascular Behçet’s disease with prominent arterial involvement: a case series of five patients

**DOI:** 10.3389/fimmu.2026.1857248

**Published:** 2026-06-24

**Authors:** Baoping He, Hengai Zhang, Qiang Ma, Yang Yang, Baiyu Chen, Lian Wang, Jianming Lai

**Affiliations:** 1 Department of Pediatrics, Affiliated Hospital of Inner Mongolia Medical University, Hohhot, China; 2 Department of Pediatrics, Ordos Maternal and Child Health Hospital, Ordos, China; 3 Department of Pediatrics, General Hospital of Ningxia Medical University, Yinchuan, China; 4 Beijing Department of Pediatrics, Beijing Children’s Hospital, Capital Medical University, Beijing, China

**Keywords:** arterial involvement, Behçet’s disease, case report, child, vascular involvement

## Abstract

**Background:**

Vascular Behçet’s disease (VBD) rare and severe subtype of Behçet disease characterized by vascular inflammation. Its prevalence in the pediatric population is extremely low. This case series aims to delineate the clinical features and long-term outcomes of pediatric VBD to enhance clinical recognition and optimize management strategies.

**Case presentation:**

We retrospectively reviewed five pediatric patients with VBD, all of whom were female. Arterial involvement was universal, with a subset of patients exhibiting concurrent venous disease. All patients showed a favorable response to glucocorticoids combined with immunosuppressants or biologic agents. Clinical manifestations were dominated by mucocutaneous and systemic features, including erythema nodosum, oral ulcers, genital ulcers, persistent fever, and abdominal pain. Laboratory evaluation revealed elevated inflammatory markers (C-reactive protein and/or erythrocyte sedimentation rate) in all cases. Vascular imaging confirmed the presence of vascular wall thickening and luminal stenosis. Colonoscopy at diagnosis identified multiple intestinal ulcers with surrounding mucosal hyperemia and edema in all five patients. “Clinical improvement“ was defined as resolution of symptoms and normalized inflammatory markers; “resolution” meant complete disappearance of vascular lesions; “amelioration” meant ≥50% reduction in wall thickening or stenosis. During follow-up, all patients exhibited significant clinical improvement, with vascular lesions demonstrating resolution or significant amelioration on repeat imaging; intestinal ulcers healed in the four patients who underwent follow-up colonoscopy.

**Conclusions:**

This pediatric case series highlights that VBD can affect vessels of various sizes and typically presents with a constellation of characteristic mucocutaneous and systemic symptoms. Early diagnosis and proactive vascular screening followed by combination immunosuppressive therapy were associated with favorable clinical and radiological outcomes. Causal conclusions cannot be drawn due to the lack of a control group. Increased awareness of this rare vasculopathy is critical for prompt intervention and improved patient prognosis.

## Background

Behçet’s disease (BD) is an immune-mediated chronic systemic vasculitis of unknown etiology that can affect multiple organ systems (1). The subset of BD characterized by vascular involvement is termed VBD. In adults, vascular manifestations of BD, including aneurysm formation and thrombosis, are major contributors to morbidity and mortality, thereby significantly impacting patient prognosis (2).

Data on the presentation of VBD in the pediatric population, particularly among Chinese children, remain exceedingly scarce. This knowledge gap underscores the diagnostic and management challenges associated with this rare condition. Herein, we report a case series of five Chinese pediatric patients with VBD to delineate their clinical profiles, with a particular focus on the prominent arterial involvement affecting vessels of various sizes. Our aim is to describe this pattern and emphasize the critical importance of proactive vascular screening in children with active BD to enable timely diagnosis and intervention.

## Case presentation

### General characteristics

This was a retrospective consecutive case series. Patients were enrolled from three centers using a unified data collection protocol. This case series included 5 female pediatric patients (age range, 10–15 years) with VBD. Clinical manifestations were heterogeneous, with prominent mucocutaneous, musculoskeletal, and gastrointestinal symptoms. Erythema nodosum was the most common presentation, followed by oral ulcers, abdominal pain, fever, and joint symptoms (**Table 1**). Isolated cases also exhibited genital ulcers, hematochezia, conjunctivitis, limb pain, skin breakdown, and sore throat. All patients met the 2015 Pediatric Behçet’s Disease (PEDBD) diagnostic criteria (1). Systematic vascular screening consisted of vascular Doppler ultrasound for peripheral vessels and computed tomography angiography (CTA) for large vessels. All images were independently reviewed by two senior radiologists blinded to clinical information. Follow-up duration ranged from 12 to 24 months (median 18 months).

**Table 1:** Clinical characteristics, vascular involvement, treatment, and follow-up outcomes of five pediatric patients with vascular Behçet’s disease.

Case	Age/sex	Key clinical manifestations	Major vascular involvement	Treatment regimen	Onset to Dx (mo)	Tx to vascular improvement (mo)	Follow-up (mo)	Follow-up outcomes
1	14/F	Abdominal pain; joint swelling/pain; upper limb pain; erythema nodosum	Left subclavian artery (wall thickening, stenosis)	Glucocorticoids + Thalidomide + Adalimumab	3	12	24	Inflammatory markers (CRP, ESR) normalized; vascular ultrasound: resolution of vascular lesions; colonoscopy: healing of intestinal ulcers; no complications
2	10/F	Recurrent oral ulcers; genital ulcers; fever	Descending aorta, abdominal aorta (wall thickening, irregular margin, mild stenosis)	Glucocorticoids + Thalidomide + Adalimumab	2	18	24	Inflammatory markers (CRP, ESR) normalized; enhanced CT: persistent but improved aortic wall thickening; no thrombosis; colonoscopy: healing of ileal ulcers
3	14/F	Erythema nodosum; arthritis; skin breakdown	Bilateral lower extremity arteries/veins (femoral, popliteal, anterior/posterior tibial arteries/veins: wall thickening, stenosis); superficial veins of left lower extremity	Glucocorticoids + Azathioprine	4	12	18	Inflammatory markers (CRP, ESR) normalized; vascular ultrasound: resolution of vascular lesions within 1 year; colonoscopy: healing of ileal ulcers; no intestinal stenosis
4	11/F	Lower limb pain; erythema nodosum; abdominal pain; hematochezia; oral ulcers; conjunctivitis	Left subclavian a.; bilateral femoral a.; bilateral anterior/posterior tibial a. (wall thickening, stenosis)	Glucocorticoids + Cyclophosphamide	3	12	18	Inflammatory markers (CRP, ESR) normalized; vascular ultrasound: resolution of vascular lesions within 1 year; colonoscopy: healing of intestinal ulcers (ileum, cecum, sigmoid colon, rectum)
5	15/F	Oral ulcers; fever; sore throat; erythema nodosum	Abdominal aorta, carotid a., axillary a. (segmental thickening, stenosis, occlusion); femoral/iliac vein thrombosis	Methylprednisolone pulse + Cyclophosphamide + Thalidomide + Adalimumab	6	12	24	Inflammatory markers (CRP, ESR) normalized; vascular ultrasound: resolution of vascular lesions within 1 year; no new thrombosis; ileostomy closure at 18 months; stable condition during follow-up (no colonoscopy recheck)

Vascular imaging demonstrated arterial involvement in all patients, with lesions affecting large, medium, and small arteries throughout the body, including the aorta, subclavian artery, femoral artery, and peripheral limb arteries (**Table 1**). The predominant radiological findings were vascular wall thickening and luminal stenosis. A subset of patients had concurrent venous involvement, manifesting as bilateral lower extremity venous wall lesions and/or femoral/iliac venous thrombosis; no isolated venous disease was observed in the cohort. Glucocorticoids were initiated at 1–2 mg/kg/day and tapered gradually; severely ill patients received methylprednisolone pulse therapy at 10–30 mg/kg/day.

All patients received glucocorticoids as the primary therapy, with individualized combination regimens including conventional immunosuppressants (thalidomide, azathioprine, cyclophosphamide) and/or anti-tumor necrosis factor-α (anti-TNF-α) biologic agents (adalimumab). Methylprednisolone pulse therapy was administered to the patient with severe disease, followed by maintenance oral glucocorticoids. During follow-up, all patients achieved normalization of inflammatory markers (CRP and ESR), with significant improvement or complete resolution of vascular lesions on repeat imaging. Intestinal ulcers identified on colonoscopy healed in all patients, without serious gastrointestinal complications such as intestinal stenosis (**Table 1**). No severe adverse events were documented during follow-up.

### Detailed description of representative cases

Among the cohort, two cases were particularly representative of the clinical heterogeneity and severity of pediatric VBD. A 15-year-old patient (Case 5) presented with oral ulcers, fever, sore throat, and erythema nodosum, and exhibited the most extensive vascular involvement: segmental wall thickening, stenosis, and occlusion of the abdominal aorta, carotid artery, and axillary artery, accompanied by femoral and iliac venous thrombosis. This patient received aggressive methylprednisolone pulse therapy combined with cyclophosphamide, thalidomide, and adalimumab. On follow-up vascular ultrasound, complete resolution of vascular lesions was noted, and the ileostomy was successfully closed 18 months post-treatment; the patient maintained stable clinical status thereafter.

The 10-year-old patient (Case 2), the youngest in the series, presented with recurrent oral and genital ulcers and fever, with isolated large-artery involvement limited to the descending and abdominal aorta (characterized by wall thickening, irregular margins, and mild luminal stenosis). Treated with glucocorticoids combined with thalidomide and adalimumab, this patient achieved normalization of inflammatory markers; enhanced CT re-evaluation demonstrated improvement in aortic wall thickening, with no thrombosis formation and complete healing of ileal ulcers (**Figure 1**). These findings reflect the favorable response of pediatric VBD to early standardized immunomodulatory therapy, even in the setting of large-vessel involvement.

**Figure 1:**
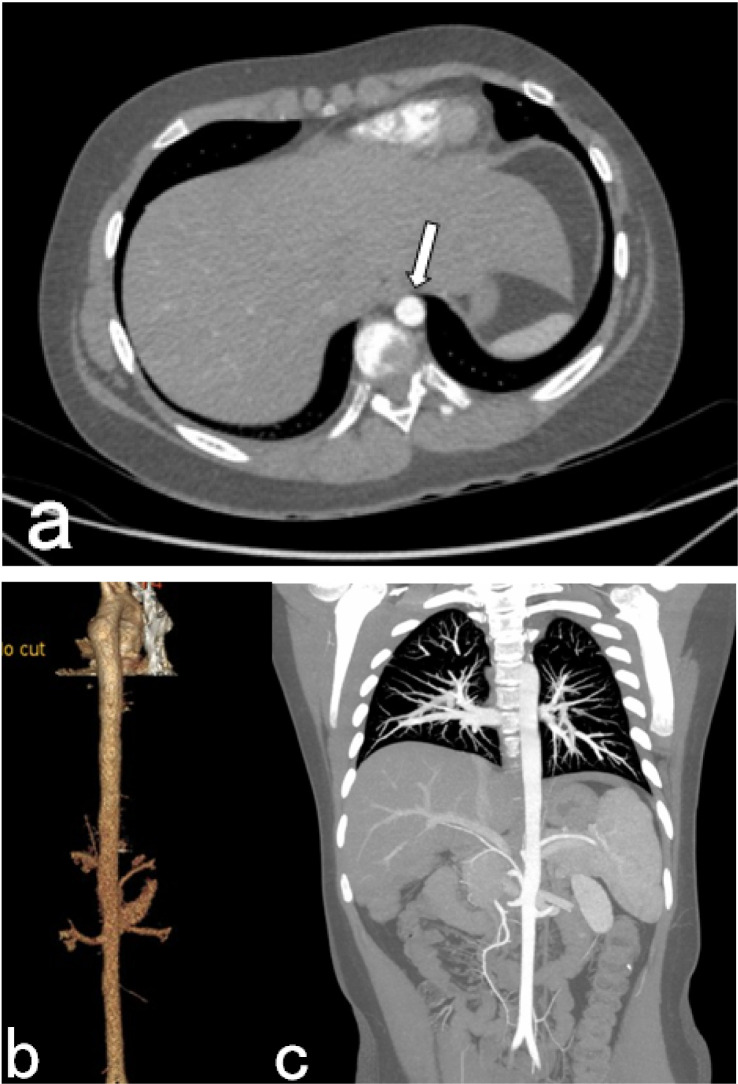
Imaging findings of arterial vascular involvement in a representative pediatric patient with vascular Behçet’s disease (Case 2). **(A)** Axial contrast-enhanced computed tomography (CT) image (arterial phase) showing localized thickening and an irregular, rough margin of the abdominal aortic wall (white arrow). **(B)** Three-dimensional CT angiography reconstruction demonstrating the extent of aortic involvement. **(C)** Coronal CT image revealing uneven wall thickness involving the descending and abdominal aorta (arterial lesions) (arrowheads).

## Discussion

### Summary of characteristics of this case series

This case series included 5 female pediatric patients with vascular Behçet’s disease, aged 10–15 years. The dominant vascular feature was extensive involvement of large, medium, and small arteries in all patients. A subset had concurrent venous involvement, but no isolated venous disease was observed. No aneurysms or cardiac involvement—common findings in adult patients—were identified in this cohort; one patient (Case 5) exhibited arterial occlusion.

Notably, none of the patients exhibited typical limb ischemic symptoms or related positive physical findings attributable to vascular stenosis or occlusion at admission. All patients presented with heterogeneous mucocutaneous, musculoskeletal, and gastrointestinal symptoms. Colonoscopy confirmed multiple intestinal ulcers in each case, and at least one inflammatory marker was elevated in all patients, consistent with active disease.

All patients received glucocorticoid-based combination therapy with individualized immunosuppressants or anti-TNF-α biologic agents. During follow-up, all achieved favorable outcomes, including normalization of inflammatory markers, improvement or resolution of vascular lesions, and healing of intestinal ulcers, without severe complications.

### Comparison with adult and pediatric literature

BD is a systemic inflammatory disorder characterized by recurrent oral ulcers, genital ulcers, and uveitis, with potential involvement of multiple organ systems including the skin, joints, gastrointestinal tract, and central nervous system (1, 2). The presence of vascular manifestations defines the subtype known as VBD. In international cohort studies, the proportion of pediatric BD patients with vascular involvement has been reported to range from 11.5% to 11.6% (3, 4), with venous lesions classically representing the predominant pattern. However, population-based prevalence data specific to Chinese children remain lacking. The arterial-predominant pattern may represent a distinct pediatric phenotype of VBD. Differences in disease stage, ethnicity, cohort characteristics, or referral patterns may contribute to this observation.

In the present series, vascular lesions were characterized by the absence of aneurysms or cardiac involvement—complications commonly described in adult VBD. While arterial occlusion was observed in one patient (Case 5), this finding was exceptional in our cohort. Pediatric VBD in the present series was characterized by a striking predominance of arterial involvement, a pattern that contrasts with the classic venous predominance reported in many international cohorts. This discrepancy may be attributed to a combination of factors, including ethnic and genetic heterogeneity, differences in disease activity at presentation, the exclusively female composition of our cohort, and variations in disease duration at the time of vascular evaluation.

This proinflammatory milieu may preferentially affect arterial structures in pediatric patients with immature vascular homeostasis. Ethnic and genetic factors may also contribute to the distinct arterial-predominant phenotype observed in our Chinese cohort. Several immunological hypotheses may explain the arterial-predominant phenotype in this pediatric cohort. First, developing arteries in children exhibit higher expression of endothelial adhesion molecules (VCAM-1, ICAM-1) and increased sensitivity to TNF-α-mediated inflammation, rendering them more vulnerable to neutrophilic infiltration and vascular remodeling (5, 6). Second, the absence of aneurysm formation may reflect age-dependent differences in matrix metalloproteinase (MMP) activity or vascular smooth muscle cell responses (7, 8). Third, the exclusively female cohort raises the possibility that estrogen modulates Th1/Th17 polarization or neutrophil extracellular trap (NET) formation, favoring arterial involvement (9, 10). While these hypotheses require direct validation, they provide a framework for future mechanistic studies in pediatric VBD.

The pathogenesis of VBD involves neutrophil hyperactivation, endothelial dysfunction, hypercoagulability, and excessive proinflammatory cytokine production. Of note, overexpression of tumor necrosis factor (TNF) plays a central pathogenic role. Neutrophils generate excessive reactive oxygen species (ROS) via NADPH oxidase, promoting fibrinogen oxidation and subsequent thrombogenesis (11–13).

Cardiac involvement represents a severe complication of adult BD, affecting the myocardium, valves, pericardium, and conduction system, and is associated with significant mortality and morbidity (14–16). In our pediatric cohort, no cardiac involvement was identified, suggesting that cardiac complications may be less common or occur later in pediatric-onset VBD.

In adult VBD, vascular lesions can affect large, medium, and small arteries and veins, with aortic involvement frequently leading to occlusion or aneurysm formation (11, 12, 17). In our series, arterial involvement was extensive and involved the descending aorta, abdominal aorta, subclavian artery, femoral artery, and peripheral small arteries. Most patients demonstrated multisegmental, multilevel arterial involvement, consistent with a systemic vasculitic process.

Previous adult studies report that venous involvement (mainly thrombosis) accounts for approximately 88% of cases, while arterial lesions typically manifest as stenosis, occlusion, or aneurysm (11, 13, 18). By contrast, our pediatric patients all had arterial involvement, with only mild concurrent venous thrombosis in two cases and no isolated venous disease. The predominant lesions were vascular wall thickening and stenosis, with no aneurysms observed in any patient. Notably, one patient had splenic infarcts, indicating the need for careful screening for end-organ ischemia even in the absence of severe vascular structural damage. These findings collectively support a distinct arterial-predominant phenotype in pediatric VBD that differs significantly from adult disease.

### Clinical implications

VBD is a rare and severe complication of active Behçet’s disease. It may involve arteries and veins of all calibers. Our findings suggest that arterial involvement may be a prominent feature in some pediatric VBD patients, contrasting with the venous predominance often described in adults. Although pathological vascular changes may be relatively mild in children, VBD can still be complicated by venous thrombosis and solid organ infarction (e.g., splenic infarcts in Case 5), warranting heightened clinical vigilance.

Vascular involvement in children with active BD may occur in the absence of typical ischemic symptoms or positive physical findings, which can lead to delayed or missed diagnosis if clinical evaluation and routine physical examination alone are used. All 5 patients in this series were identified through systematic vascular screening. Pediatric VBD is associated with favorable outcomes following early diagnosis and prompt treatment with glucocorticoids combined with immunosuppressants or biologic agents, with most vascular lesions showing complete reversal.

A proposed screening workflow includes: 1) Confirm BD diagnosis; 2) Systemic evaluation; 3) Vascular ultrasound and CTA; 4) Follow-up imaging and laboratory tests. Therefore, clinicians should maintain a high index of suspicion for pediatric VBD and implement systematic vascular imaging evaluations, including vascular Doppler ultrasound and computed tomography angiography, as a routine component of assessment for children with active BD. Such an approach enables early identification of vascular involvement and provides critical support for timely intervention and improved patient prognosis.

This study has several limitations, including a small retrospective sample size, lack of a control group, relatively short follow-up duration, and narrow study scope. Although data were collected from three hospitals, this was not a formal multicenter study, limiting the generalizability of the results. Furthermore, this study cannot provide definitive conclusions regarding long-term prognosis or allow in-depth investigation of the underlying disease mechanisms. The all-female cohort may limit the generalizability of the arterial-predominant pattern. This study lacks patient-specific immunological profiling and biomarker analysis. Patient perspectives were not collected in this retrospective study. The all-female distribution may suggest sex-related susceptibility, hormonal effects, or referral bias. A direct comparison between arterial-predominant and isolated venous involvement was not feasible in this small case series. Future studies focusing on clinical disparities, risk factors, and immunological mechanisms underlying arterial involvement in pediatric VBD are warranted.

## Conclusion

This pediatric case series demonstrates that VBD can involve arteries and veins of various sizes in children, with mucocutaneous lesions and systemic symptoms as the primary clinical presentations. Proactive vascular screening in children with active Behçet disease is key to early diagnosis. Early diagnosis and combination immunosuppressive therapy were associated with favorable outcomes in this case series. Causal conclusions cannot be drawn due to the lack of a control group. Whether this arterial pattern represents a distinct pediatric phenotype requires further validation in larger cohorts. Enhancing clinicians’ awareness of this rare pediatric vasculopathy is fundamental to timely intervention and optimized patient prognosis.

## Data Availability

The original contributions presented in the study are included in the article/**Supplementary Material**. Further inquiries can be directed to the corresponding authors.
