# Supplementary material for: Case report: Pediatric vascular Behçet’s disease with prominent arterial involvement: a case series of five patients
Source: Front Immunol. 2026 Jun 24;17:1857248. doi: 10.3389/fimmu.2026.1857248 (PMC13343165; doi:10.3389/fimmu.2026.1857248)
Supplement: Supplementary file 1 [file DataSheet1.pdf]

| Topic | Item | Checklist item description | Location |
|---|---|---|---|
| Title | 1 | The diagnosis or intervention of primary focus followed by the words "case report" | **Title line / Title page** |
| Key Words | 2 | 2 to 5 key words that identify diagnoses or interventions in this case report, including "case report" | **Keywords section** |
| Abstract (no references) | 3a | Introduction: What is unique about this case and what does it add to the scientific literature? | **Abstract section** |
| | 3b | Main symptoms and/or important clinical findings | **Abstract section** |
| | 3c | The main diagnoses, therapeutic interventions, and outcomes | **Abstract section** |
| | 3d | Conclusion-What is the main "take-away" lesson(s) from this case? | **Abstract section** |
| Introduction | 4 | One or two paragraphs summarizing why this case is unique (may include references) | **Introduction section** |
| Patient Information | 5a | De-identified patient specific information | **Case presentation** |
| | 5b | Primary concerns and symptoms of the patient | **Table 1** |
| | 5c | Medical, family, and psycho-social history including relevant genetic information | **Not applicable** |
| | 5d | Relevant past interventions with outcomes | **Table 1** |
| Clinical Findings | 6 | Describe significant physical examination (PE) and important clinical findings. | **Case presentation; Table 1** |
| Timeline | 7 | Historical and current | **Table 1** |

| Topic | Item | Checklist item description | Location |
|---|---|---|---|
| | | information from this episode of care organized as a timeline | |
| Diagnostic Assessment | 8a | Diagnostic testing (such as PE, laboratory testing, imaging, surveys) | Case presentation; Figure 1 |
| | 8b | Diagnostic challenges (such as access to testing, financial, or cultural) | Not applicable |
| | 8c | Diagnosis (including other diagnoses considered) | Case presentation |
| | 8d | Prognosis (such as staging in oncology) where applicable | Table 1 |
| Therapeutic Intervention | 9a | Types of therapeutic intervention (such as pharmacological, surgical, preventive, self-care) | Table 1 |
| | 9b | Administration of therapeutic intervention (such as dosage, strength, duration) | Table 1 |
| | 9c | Changes in therapeutic intervention (with rationale) | Table 1 |
| Follow-up and Outcomes | 10a | Clinician and patient-assessed outcomes (if available) | Table 1 |
| | 10b | Important follow-up diagnostic and other test results | Table 1 |
| | 10c | Intervention adherence and tolerability (How was this assessed?) | Case presentation |
| | 10d | Adverse and unanticipated events | Case presentation |
| Discussion | 11a | A scientific discussion of the strengths AND limitations associated with this case report | Discussion section |

| Topic | Item | Checklist item description | Location |
|---|---|---|---|
| | 11b | Discussion of the relevant medical literature with references | **Discussion section** |
| | 11c | The scientific rationale for any conclusions (including assessment of possible causes) | **Discussion section** |
| | 11d | The primary "take-away" lessons of this case report (without references) in a one-paragraph conclusion | **Discussion section** |
| Patient Perspective | 12 | The patient should share their perspective in one to two paragraphs on the treatment(s) they received | **Not applicable** |
| Informed Consent | 13 | Did the patient give informed consent? Please provide if requested | **Yes** |
